# Comparative analysis of microbiome in coronal and root caries

**DOI:** 10.1186/s12903-024-04670-3

**Published:** 2024-07-31

**Authors:** Tadamu Gondo, Noriko Hiraishi, Azusa Takeuchi, David Moyes, Yasushi Shimada

**Affiliations:** 1 Cariology and Operative Dentistry, Graduate School of Medical and Dental Sciences, Tokyo Medical and Dental University, 1-5-45, Yushima, Bunkyo-Ku, Tokyo, 113-8549 Japan; 2 Centre for Host-Microbiome Interactions, Faculty of Dentistry, Oral and Craniofacial Sciences, King’s College London, London, SE1 1UL UK

**Keywords:** Cariology, Microbiome, Caries, 16S rRNA, NGS, ICDAS

## Abstract

**Background:**

The global rise in the elderly population has increased the prevalence of root caries. *Streptococcus mutans*, *Lactobacilli* and *Actinomyces* are considered the primary pathogens of dental caries in culture-based studies. This study aimed to investigate bacterial profiles in coronal and root caries lesions and determine the association of specific bacterial genera at each site.

**Methods:**

Dentine samples from carious lesions were collected from 22 extracted teeth using an excavator. Microbial DNA was extracted from the samples using a protocol developed for this study. 16S rRNA gene amplicon sequencing was employed for microbial analysis. PCR amplification targeted the V3-V4 region of the bacterial 16S rRNA, and the amplicon sequencing used an Illumina MiSeq system (2 × 300 bp paired-end reads). Statistical analysis was performed by the Phyloseq and DESeq2 packages in R.

**Results:**

In coronal caries, *Olsenella*, *Lactobacillus* and *Prevotella* were the most prevalent genera, comprising approximately 70% of the microbiome community. In the root caries, however, although *Olsenella, Prevotella* and *Lactobacillus* remained the dominant genera, they accounted for only half of the microbiome community. This study identified significant differences in alpha diversity indices between the coronal and root caries. LEfSE analysis revealed several unique genera in each caries lesion.

**Conclusion:**

The microbiome of root caries lesions was richer and more complex than the coronal caries microbiota. The results suggest that lesion-related variations in the oral microflora may be detected in carious dentine.

**Supplementary Information:**

The online version contains supplementary material available at 10.1186/s12903-024-04670-3.

## Backgrounds

The number of elderly people is rising worldwide. Even though individuals remain healthy until late life and have many residual teeth, the prevalence of root caries increases with age, which has raised serious concerns [1, 2]. Gingival recession in the elderly leads to increased root surface exposure, and root caries is further associated with problems specific to the elderly, such as decreased saliva, dry mouth, and systemic disease [3]. According to a recent systematic review, age, poor oral hygiene, gingival recession, lower socio-economic status, and use of tobacco have a higher risk of root caries [4]. Prevention and management of root caries is challenging for individuals with poor oral hygiene or certain medical conditions. Proper oral hygiene may be even more difficult for the elderly, especially those with physical limitations or certain medical conditions. Dental professionals highly recommend regular dental check-ups and cleanings to detect early signs of root caries. Some studies have also been conducted to explore effective prevention and management strategies by applying fluoride, including silver diamine fluoride [5, 6]. However, there is currently insufficient evidence regarding the prevention of root surface caries. Thus, the aetiology of these lesions needs to be thoroughly investigated and a practical approach to their prevention must be established.

*Streptococcus mutans* is regarded as a pathogen in dental caries [7]. However, there has been much debate over the aetiology of caries. Various studies exploring the microbial causes of caries have employed culture-based methodologies [8, 9]. However, because numerous bacteria cannot be cultivated outside the human milieu, culture-based approaches do not identify all types of bacteria [10]. Recent molecular biology-based research, in contrast to traditional culture-based research, revealed the full diversity of microbial communities of the oral microflora. This approach was applied to investigate the bacterial profiles of the microflora of root caries in the elderly [11, 12]. Preza et al. suggested that the microbial communities within the oral cavity have varied complexity depending on the site [11].

High throughput DNA sequencing technology and subsequent bioinformatics pipelines have advanced significantly in recent years. Compared to Sanger sequencing, next-generation gene sequencing (NGS) provides massive and high-throughput base sequence analysis, allowing for detailed and sensitive bioinformatic analyses of complex microbial communities. NGS techniques have been applied to investigate the microbiome of dental caries [13], although the samples were often collected from a biofilm that covered or surrounded the caries lesions. To our knowledge, there are a limited number of studies that sampled from caries dentine [12, 14–17]. Concerning root caries, two studies have examined the microflora by sampling affected dentine [12, 15].

The progression of crown and root caries is accompanied by acid demineralisation and protein digestion [18], but the local environment may be different. Coronal surfaces are exposed to saliva, whilst root surfaces are exposed to gingival crevicular fluid. Both fluids have similarities, including buffering effects and ion composition, but there are some differences in immune components [19]. The gingival crevicular fluid contains leukocytes, IgG, IgM and IgA as antimicrobial components, whilst saliva contains sIgA, histatins and lactoferrin [19]. Thus, the aetiology may differ due to the different local environments. However, no research has been conducted to compare the microbial communities between coronal and root caries.

This study aims to investigate the microbial variations of coronal and root caries. To better understand the factors that influence the development and progression of each caries lesion, it is important to identify the unique microbial profile associated with each caries morphology. This study provides insights into therapeutic and preventive approaches to improve the oral health of the ageing population. The null hypothesis was that the microbiome in both caries lesions would not have a different composition.

## Methods

### Study population and sample collection

The study design and sample collection were approved by the Ethical Committee of the Tokyo Medical and Dental University Graduate School of Medical and Dental Science (approval number: D2021-034 ). Teeth with severely progressed dental caries and periodontal disease, which were diagnosed as indicated for extraction, were used as samples. Informed consent was obtained from donating subjects with respect to the use of human tissues for research. Teeth was collected from August 2021 to August 2022. Patient were excluded if they had severe systemic diseases, received systemic or local administration of antibiotics within the past 3 months. A total of 22 extracted teeth with dental caries (12 coronal caries and 10 root caries) were included in the study. There were composite resin fillings in the sample (Coronal: 2/12 Root: 7/10), but the fillings were located away from cavities. The extracted teeth were obtained from 11 females and 11 males, with an age range of 27-86 years and a median age of 51 years. Following extraction, the tooth was immediately frozen and preserved until carious dentine sample collection. Before the sample collection, an X-ray photo was taken to validate the presence of caries. All samples were classified according to the ICDAS (International Caries Detection and Assessment System) Code. The ICDAS diagnosis concordance was performed by three dental professionals. The tooth was then defrosted and cleaned of blood and plaque with saline water. The soft tissues of the teeth and the superficial layers of carious lesions were carefully removed with sterile dental instruments. If the enamel remained over the carious dentin, the enamel was gently crushed with a sterile chisel. Caries-affected softened dentine samples were collected using a sterile spoon excavator 8 to 10 times and placed in a tube containing preservation solution (100 mM Tris–HCl (pH 9) (Nippon Gene, Tokyo, Japan), 40 mM EDTA (Nippon Gene, Tokyo, Japan), 4 M guanidine thiocyanate (Fujifilm-Wako, Tokyo, Japan)) at -20°C until DNA extraction. One individual performed all sample collections.

### DNA extraction

Microbial DNA was extracted from the samples using a protocol developed for this study. Using a 0.5 M EDTA (Nippon Gene, Tokyo, Japan) solution and a thermomixer, the obtained dentine sample was decalcified for 72 h at 37 °C. Following decalcification, ceramic beads (φ1.4 mm, Qiagen, Valencia, CA, USA) were added to the collection tube, and then the mixture was homogenised for 5 min using a TissueLyser LT (Qiagen, Valencia, CA, USA) at 50 Hz. The ISOSPIN Faecal DNA Kit (Nippon Gene, Tokyo, Japan) was subsequently used to extract microbiome DNA.

### 16S rRNA gene amplicon sequencing

PCR amplification targeted the V3-V4 region of the bacterial 16S rRNA gene was performed using forward and reverse primers containing the adapter sequence with barcode index; 341F(5’-ACACTCTTTCCCTACACGACGCTCTTCCGATCT-NNNNN- CCTACGGGNGGCWGCAG-3’) and 381R (5’- GTGACTGGAGTTCAGACGTGTGCTCTTCCGATCT-NNNNN-GACTACHVGGGTATCTAATCC-3’). Library construction and the amplicons sequencing using an Illumina MiSeq system (2 × 300 bp paired-end reads) were performed at Bioengineering Lab. Co. Ltd. (Kanagawa, Japan).

### Data analysis and taxonomy assignments

The raw sequence data was processed using the FASTX-Toolkit (ver. 0.0.14). Only read sequences that matched the primer sequences exactly were extracted using the fastx_barcode_splitter tool. Primer sequences were removed from the extracted reads with fastx_trimer of FASTX-Toolkit. Sequences with a quality value of less than 20 were then removed using sickle (ver. 1.33). Sequences with a length of less than 130 bases and their paired sequences were removed and discarded. The paired-end reads were merged using FLASH (ver. 1.2.11). DADA2 in QIIME2 was used for the removal of chimeras and amplicon sequence variant (ASV) inference. Amplicons were grouped into amplicon sequence variants (ASVs) with 100% sequence similarity. The percentage of input non-chimeric was 67.8 – 88.09. Subsequently, taxonomy was assigned using the Silva 138 rRNA database (silva-138–99-nb-classifier) using the Naïve Bayesian Classifier algorithm default in DADA2. The confidence level of 0.7 (default setting) was used for the taxonomy assignment.

### Statistical analysis

Statistical analysis used R (RStudio version; 4.1.2, RStudio, Boston, MA, USA). *Phyloseq* and *DESeq2* packages were used to perform bacterial community analysis. Chao1, Shannon and Simpson indexes were compared using the Wilcoxon rank-sum test. PERMANOVA test was performed to compare the beta diversity index. A principal coordinates analysis (PCoA) plot was generated to demonstrate the compositional difference. Linear discriminant analysis effect size (LEfSE) analysis revealed microbiome characteristics at the genus level. For all tests, *p*-values of < 0.05 were considered statistically significant.

## Results

### Subject population

Demographic characteristics, ICDAS code and representative X-ray photos are shown in Table 1 and Appendix 1. The coronal caries samples were classified into Code 4, Code 5 and Code 6, and all root caries samples were classified into Code 1. The kappa value of ICDAS code classication was 0.825.

**Table 1:** Demographic characteristics of patients by group and ICDAS code

**Characteristics**	**Coronal**, *N* = 12^1^	**Root**, *N* = 10^1^	***p*-value**^2^
**Sex**			0.4
Female	5 (42%)	6 (60%)	
Male	7 (58%)	4 (40%)	
**Age**	44 (13)	70 (18)	0.001
**ICDAS**			
Code1	-	10 (100%)	
Code4	3 (25%)		
Code5	4 (33%)		
Code6	5 (42%)		

^1^n (%); Mean (SD)

^2^Pearson's Chi-squared test; Welch Two Sample t-test; Fisher's exact test

### Microbial profile based on 16S rRNA gene sequencing

**Fig. 1:**
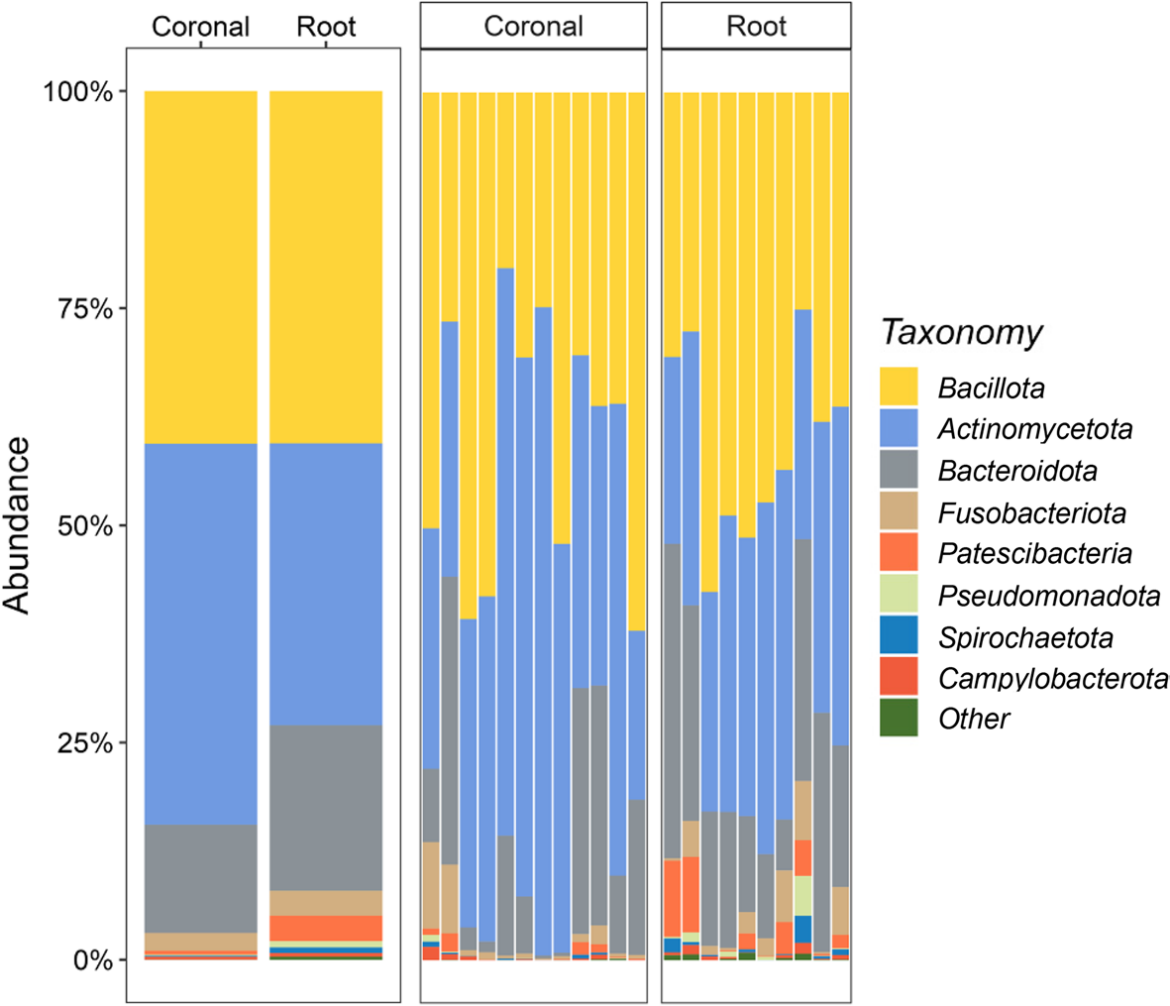
Relative abundance of major bacterial phyla. Composition of the oral microbial profile at the phylum level as the percentage of relative abundance by the caries groups. The mean of the relative abundance by each group was shown on the left

**Fig. 2:**
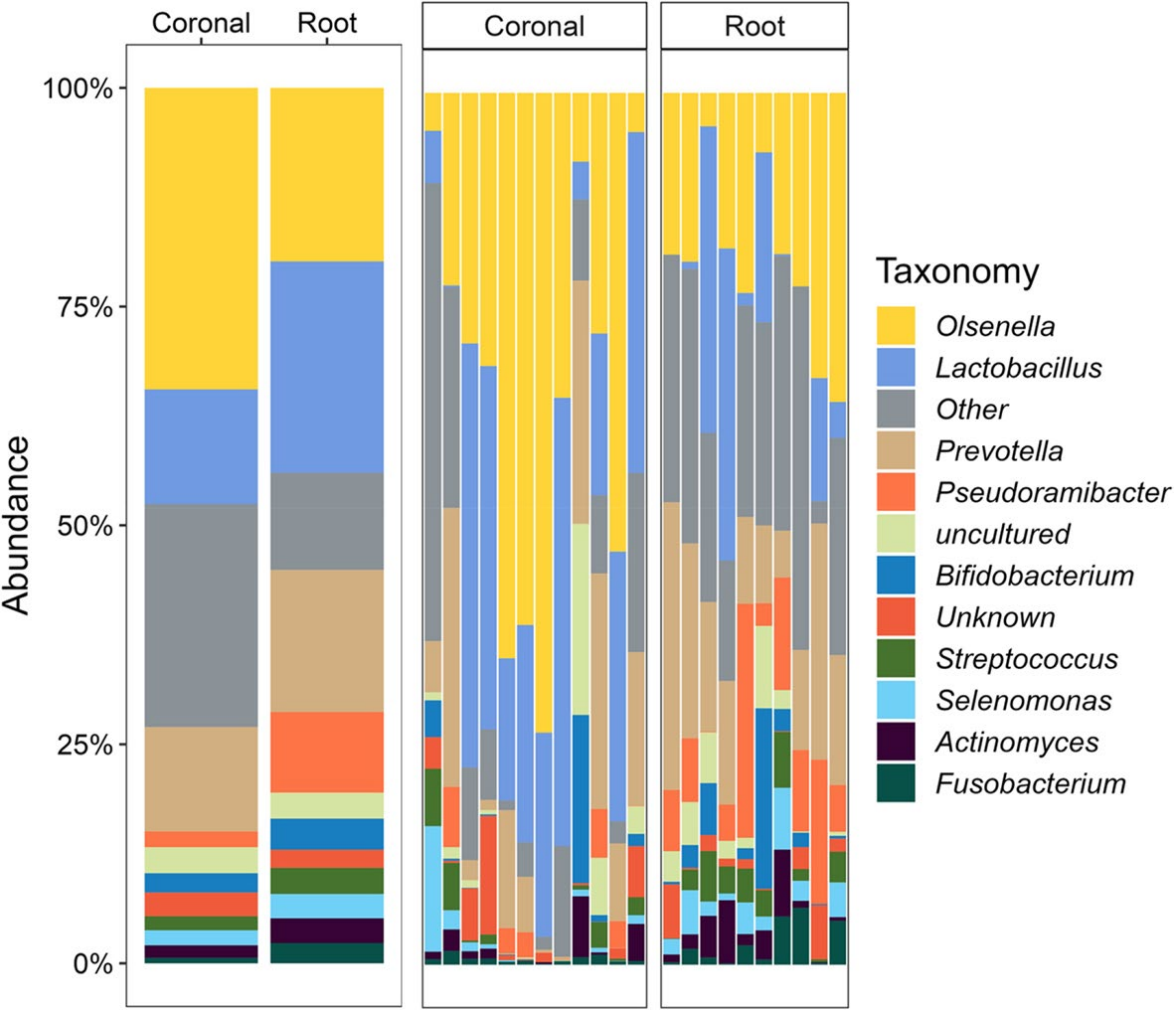
Relative abundance of major bacterial genera. Composition of the oral microbial profile at the genus level as the percentage of relative abundance by the caries group. The mean of the relative abundance by each group was shown on the left

16S rRNA sequencing was used to determine the microbial composition of each caries lesion. According to relative abundance, the most common phyla in the coronal caries group were *Actinomycetota* (43.9%), *Bacillota* (40.6%) and *Bacteroidota* (12.5%), whereas the most common phyla in the root caries group were *Bacillota* (40.6%), *Actinomycetota* (32.5%) and *Bacteroidota* (19.1%) (Fig. 1). At the genus level (Fig. 2), the three most prevalent genera in the coronal sample were *Olsenella* (34.5%), *Lactobacillus* (25.5%) and *Prevotella* (11.9%). These three genera accounted for approximately 70% of the microbiome. Similar to the coronal sample, the most frequent genera in the root sample were *Olsenella* (19.8%), *Prevotella* (16.2%) and *Lactobacillus* (11.1%). The rest of the genera of both caries groups are similar, although *Pseudoramibacter*, *Fusobacterium* and *Actinomyces* were more abundant in the root caries group. Notably, the prevalence of *Streptococcus* genera including *S. mutans* was only 1.6% in the coronal sample and 3.0% in the root sample.

### The diversity of microbiome in caries lesions

**Fig. 3:**
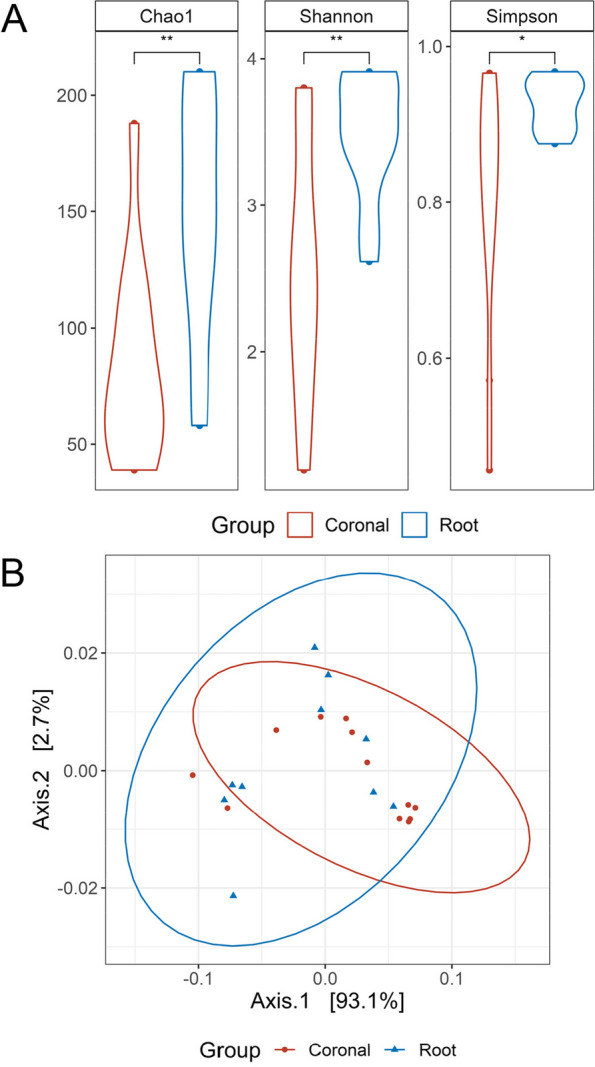
The diversity of microbiome in coronal and root caries lesions. Alpha diversity analysis using Chao 1, Shannon, and Simpson index (**A**). PCoA plot was generated to demonstrate the compositional difference. PCoA plot weighted unifrac (**B**) The significance of differences between the two groups is evaluated. *p* values < 0.05 are considered to indicate statistical significance. **: *p* < 0.01, *: *p* < 0.05

To investigate the microbial community, the alpha diversity parameters including Chao1, Shannon and Simpson indices were calculated. Each index showed significant differences in the alpha diversity between the coronal and root groups (Fig. 3). However, in the beta diversity, the PERMANOVA test showed no significant differences between the two groups with no apparent clustering in principal component analysis (Fig. 3).

### Bacterial characteristics

**Fig. 4:**
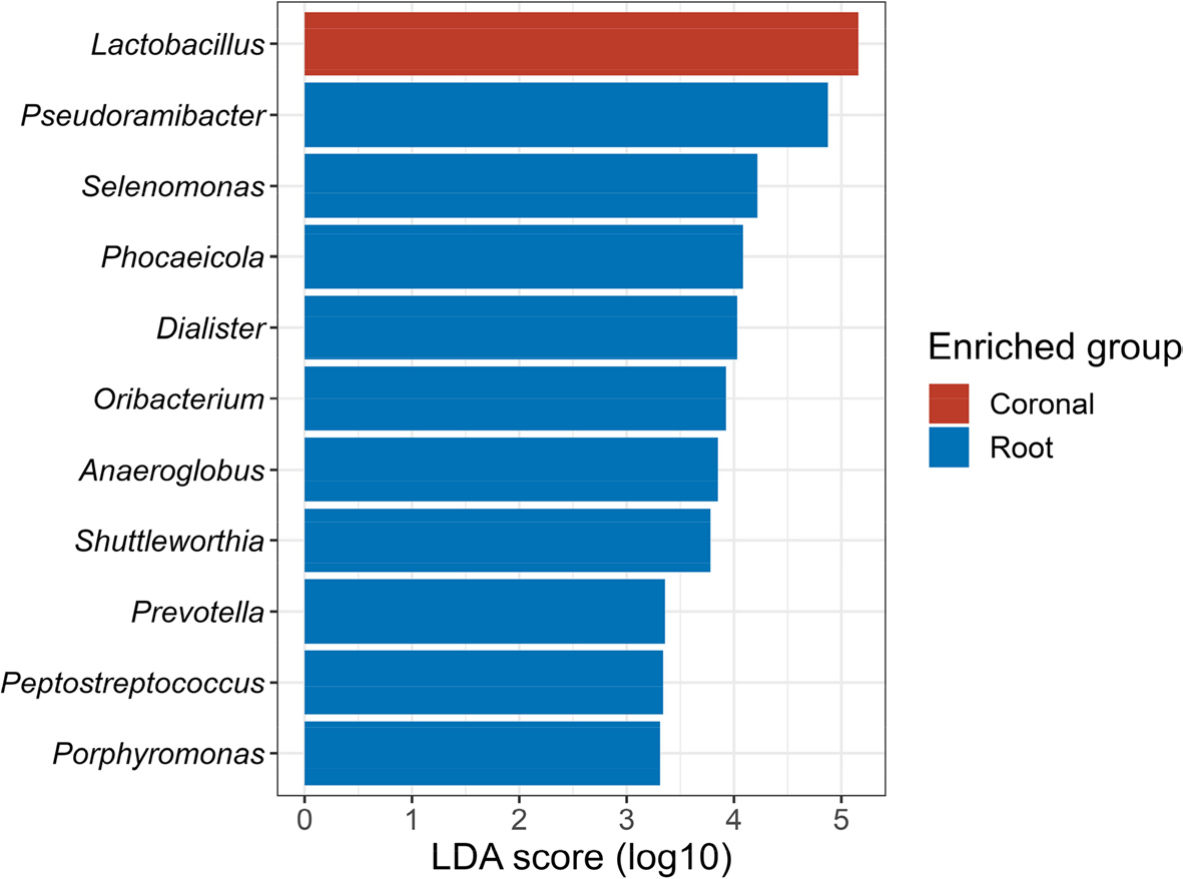
The linear discriminant analysis effect size (LEfSe). The differentially abundant genera between the coronal caries group and the root caries group. Bar plots show linear discriminant analysis (LDA) scores of each genus. The LDA scores indicate the effect sizes of each genus, and genera with an LDA score ≥ 3 are shown

Discriminative microbial features between the coronal and the root caries groups were determined by LEfSE. The LEfSE analysis of relative abundances at the genus level revealed the microbiome characteristics of each group (Fig. 4). *Lactobacillus* was identified as an enriched genus in the coronal caries group. In contrast, multiple genera were enriched in the root surface caries group–– *Pseudoramibacter*, *Selenomonas*, *Phocaeicola*, *Dialister*, *Oribacterium*, *Anaeroglobus*, *Shuttleworthia*, *Prevotella*, *Peptostreptococcus* and *Porphyromonas* genera were all abundant.

## Discussion

This study collected samples from caries-affected dentine and performed a genomic analysis. Culture-independent methods have been typically used to study oral microbiomes by collecting samples from saliva and dental plaque. However, the current study was the first attempt to characterise the microbiome associated with coronal and root caries using 16S rRNA gene sequencing, a culture-independent method. The findings rejected the null hypothesis that there would be no difference in the microbiome composition in the two caries lesion types.

When comparing the previous studies on the caries dentine microbiome, the results of the present study were similar to a previous study [14], in terms of the order of relative abundance at the phylum level. *Olsenella* genus, which has been indicated as a putative aetiological agent [12, 16], was also detected in the carious dentine samples in this study. The *Lactobacillus* genus was identified as a unique genus in the LEfSE analysis. A comprehensive microbiome profiling by shotgun metagenomic sequencing also showed that *Lactobacillus* spp. was most abundant in carious dentine samples from under 18 years of age [17].

Furthermore, the higher diversity of the microbiome in the root caries in our present study was consistent with a previous study [15]. Diversity analysis revealed that the microbiome of root caries is more rich and complex than the coronal caries microbiome, although neither group showed clear clusters. According to a previous study on dental plaque associated with severe early-childhood caries, the microbiome profile became less complex as caries progressed [20]. These results may provide an interpretation of the present results regarding the maturity of the microbiome, although there was a difference in the location of collection, i.e., plaque or carious dentine. Coronal caries usually progresses beneath the enamel as long as fermentable carbohydrates are available, acidic conditions continue, and the lesions become cavities covered by enamel overlays. As in the case of accumulated plaque, the local environment underneath the enamel becomes more anaerobic and less nutritious over time, which may lead to a reduction in microbiome diversity. The mechanisms of the reduction in microbiome diversity as caries progresses and the microbiome matures will need to be elucidated.

When dental caries occurs, the normal balance between the oral microbiome and the host is disrupted, and the equilibrium is lost. This phenomenon, called dysbiosis, primarily occurs due to the excessive growth of cariogenic bacteria, such as *S. mutans.* These species metabolise fermentable dietary sugars (such as sucrose), causing a decrease in pH levels that drives the demineralisation of tooth structure and ultimately leads to dental caries [21]. However, the results of the current study did not definitively confirm the significant presence of *Streptococcus* genera in the caries lesions. The reason for this low abundance of *Streptococcus* genera is unknown but may be related to the location from which the samples were collected. Most previous microbiological studies of caries development and pH changes have been linked to dental plaque or tooth surface biofilms. In contrast, the caries samples analysed in the current study were collected from infected dentine hard tissue in an existing developed lesion but not from the overlying plaque. In a previous 16S rRNA gene sequencing study [22], changes in bacterial profiles in caries lesions were examined in individuals with different caries degrees, where a predominance of acid-producing bacterial species other than *S. mutans* was found involved in deep caries lesions. This finding and our current results suggest that *S.mutans* may play a role in the early development of caries lesions but potentially not the primary pathogen in developed caries lesions.

The current results show that at the phylum level, the microbial profiles of both coronal and root caries lesions were similar in composition. *Bacillota*, *Actinomycetota* and *Bacteroidota* accounted for more than 90% of all phyla found. In contrast, the order of relative abundance of each genus was different when compared with the salivary microbiome of a healthy Japanese population [23, 24]. *Actinomycetota* in both caries lesions became more abundant than in healthy saliva, and *Pseudomonadota* and *Fusobacteriota* became less prevalent than in healthy saliva. Interestingly, according to Saito et al., *Neisseria* spp. belonged to the *Proteobacteria* phylum and were commonly found in healthy saliva and plaque, but the abundance of *Neisseria* decreased as caries progressed [23]. Instead of *Neisseria, Olsenella, Lactobacillus* and *Prevotella* were abundant genera in both caries lesions in the current result*. Lactobacillus* is an acidogenic/aciduric bacterial genus, whilst *Prevotella* spp. are proteolytic/amino acid-degrading bacteria [18]. In contrast, the role and function of *Olsenella* bacteria within the caries lesion remains largely undetermined. O*lsenella* has previously been found in endodontic infection following the introduction of molecular biology methods [25]. Recent studies utilising NGS techniques have reported a significant presence of *Olsenella* in dental caries lesions [12, 16]. Notably, some species of *Olsenella* produce lactic acid [26] and are peptidolytic and acid-tolerant [27]. Thus, *Olsenella* is potentially involved in the progression of dental caries. Although not typically considered a major causative pathogen of pulpal necrosis, it may play a significant role in pulpal infection.

The relative abundance of the microbiome was re-analysed to determine the relationship between the ICDAS code and the diversity of the microbiome (Appendix 2). The criteria for ICDAS Code 6 was “Extensive distinct cavity with visible dentine,” Code 5 was “Distinct cavity with visible dentine,” and Code 4 was “Underlying dark shadow from dentine with or without localised enamel breakdown.” The relative abundance of the microbiome in the Code 5 and Code 6 groups was more diverse than that of the Code 4 group. The Code 4 cavities are protected from physical interaction with the outside environment and the anaerobic degree is higher compared to Code 5 and Code 6 cavities, which may lead to exclusive overgrowth of specific microbial species within the cavities. The results of the re-analysis showed that the relative abundance of the *Olsenella* genus decreased according to the ICDAS codes. *Olsenella* species are anaerobic bacteria and have been reported to be particularly active in lactic acid production and peptide degradation [27]. Since the high abundance of Olsenella genus in the Code 4 cavities and the decrease in abundance with the progression of caries, Olsenella may contribute to the onset of dental caries and progression of early stages of caries.

According to the LEfSE analysis carried out in this study, several bacterial genera were revealed as unique or enriched within each caries lesion. In particular, the *Lactobacillus* genus was dominant in the coronal caries lesion, suggesting *Lactobacillus* spp. may play a major role in caries progression. *Lactobacillus* species produce lactate from carbohydrate fermentation, and this lactate is commonly found in active dentine cavities, where it is thought to play a key role in decalcification. Moreover, the production of lactic acid promotes a low pH environment within the caries lesion. In this low pH environment, the microbial diversity was decreased, and particular species became dominant, e.g., *Lactobacillus* and *Propionibacterium* [28]. On the other hand, some strains of *Lactobacillus* are reported to have the effect of probiotics against dental caries [29]. In order to determine the potential of probiotic therapy for caries management, more research is needed on *Lactobacillus* species with anti-cariogenic effects or on other strains that can control *Lactobacillus* behaviour. It is, therefore, necessary to focus on the entire microbiome and its metabolic activity.

There were several limitations to this study: study population, sample collection and microbiome analysis method.

i) A healthy control was not defined, since it was difficult to obtain dental hard tissue samples from intact sites with a spoon excavator. In future studies, the inclusion of a healthy control would enhance the robustness of the findings and provide a more comprehensive understanding of the microbiome differences between the coronal and root caries lesions.
ii) Oral examinations such as DMFT, plaque index and bleeding on probing were not performed in the present study. These are the best prognostic parameters for assessing oral health. The DMFT is particularly important for discussing comparative analysis of the microbiome in further research, as the DMFT is primarily used as a historical indicator of dental caries.
iii) In this study, the 16S rRNA gene sequence was employed to determine the composition of the microbiome. However, this approach was restricted to genus-level analysis and could not effectively determine the functional nature of the microbiome, i.e., metabolic pathways, etc. DNA extraction strategies and amplification of the 16S rRNA gene may influence the results of oral microbiome biodiversity profiling through amplification and sequence bias, although targeting the hypervariable regions targeting V3–V4 enables reproducible bacterial taxonomic determination. In addition, because the study only collected specimens after tooth extraction, it was not possible to determine which bacteria contributed to the development of caries and disease progression. Longitudinal studies on the oral microbiome are essential for elucidating caries development in the first instance.
iv) Another weakness of microbiome studies related to dental caries is that they limit caries pathogens to bacteria [30]. *Candida* species are opportunistic fungi that can colonise the oral cavity and are responsible for mucosal infections in immunocompromised hosts [31]. *Candida* species are also well known for extracellular matrix-forming interactions between *S. mutan*s and *C. albicans* [32]. Moreover, *Candida* species have been shown to metabolise carboxylic acid as well as the lactate produced by acidogenic bacteria [33], which leads to the elevation of the local environment pH. Therefore, further research on the caries microbiome should focus on fungi as well as bacteria, especially in the study of root caries.

## Conclusions

The microbial composition at the phylum level was similar between the root and the coronal caries lesions; however, at the genus level, the root caries lesions displayed higher diversity compared to the coronal caries lesions. *Streptococcus* spp. were low in abundance for both caries lesions. *Lactobacillus* was identified as a potential pathogen in the progression of coronal caries lesions. Meanwhile, several genera were identified as unique in the root caries lesions, although their contribution to root caries progression was not determined due to the lack of information on these species. These findings may lead to elucidating the aetiology of coronal and root caries and developing appropriate control methods for each.

### Supplementary Information

Supplementary Material 1.

## Data Availability

The sequence data have been deposited in the DDBJ Sequence Read Archive under accession number DRA017642.
