# Supplementary material for: Comparative analysis of microbiome in coronal and root caries
Source: BMC Oral Health. 2024 Jul 31;24:869. doi: 10.1186/s12903-024-04670-3 (PMC11292881; doi:10.1186/s12903-024-04670-3)
Supplement: Supplementary file 1 — Supplementary Material 1. [file 12903_2024_4670_MOESM1_ESM.docx]

Appendix 1


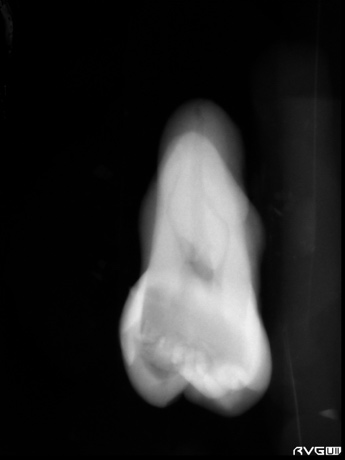

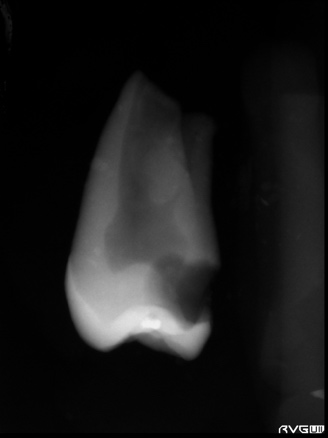

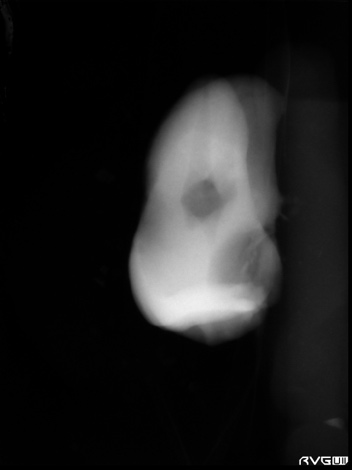

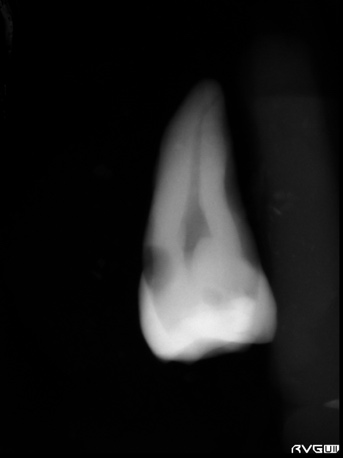


Code1

Code4

Code6

Code5

Representative X-ray photos of each ICDAS code

Appendix 2


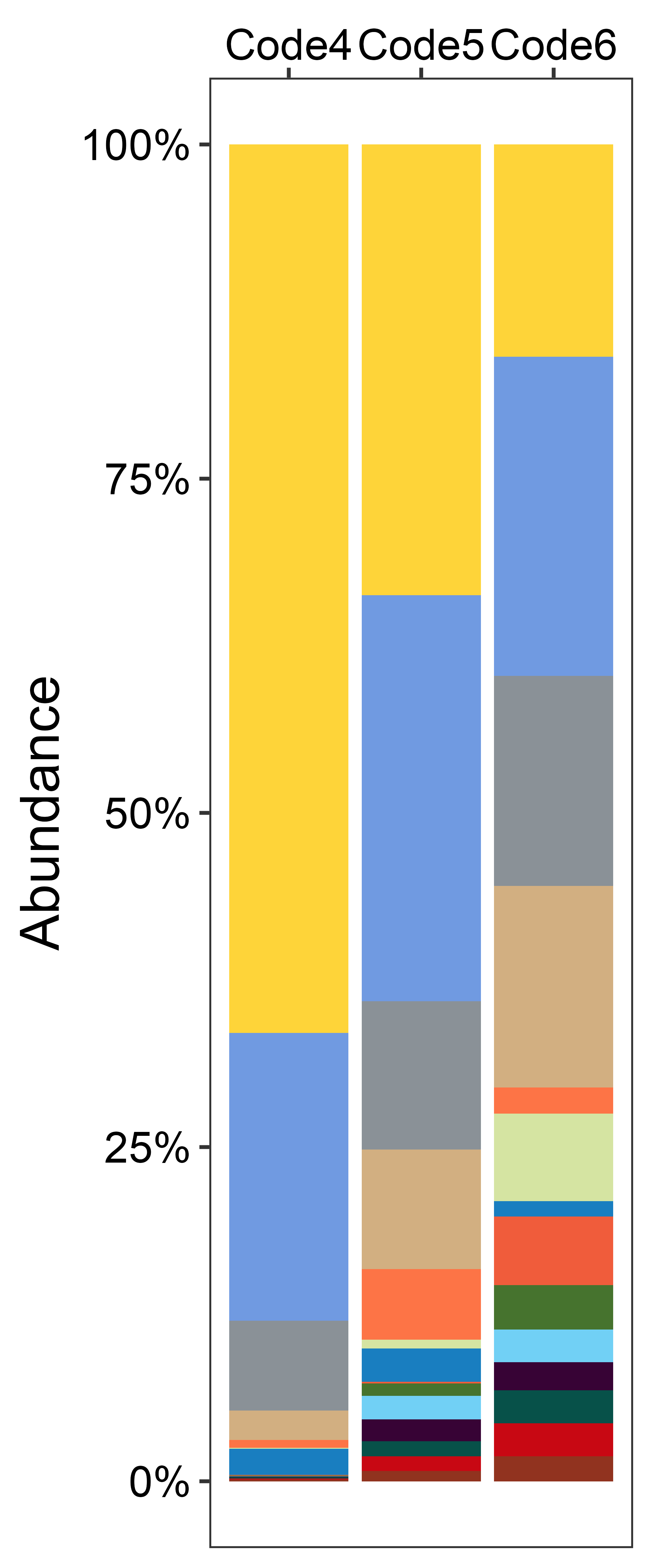

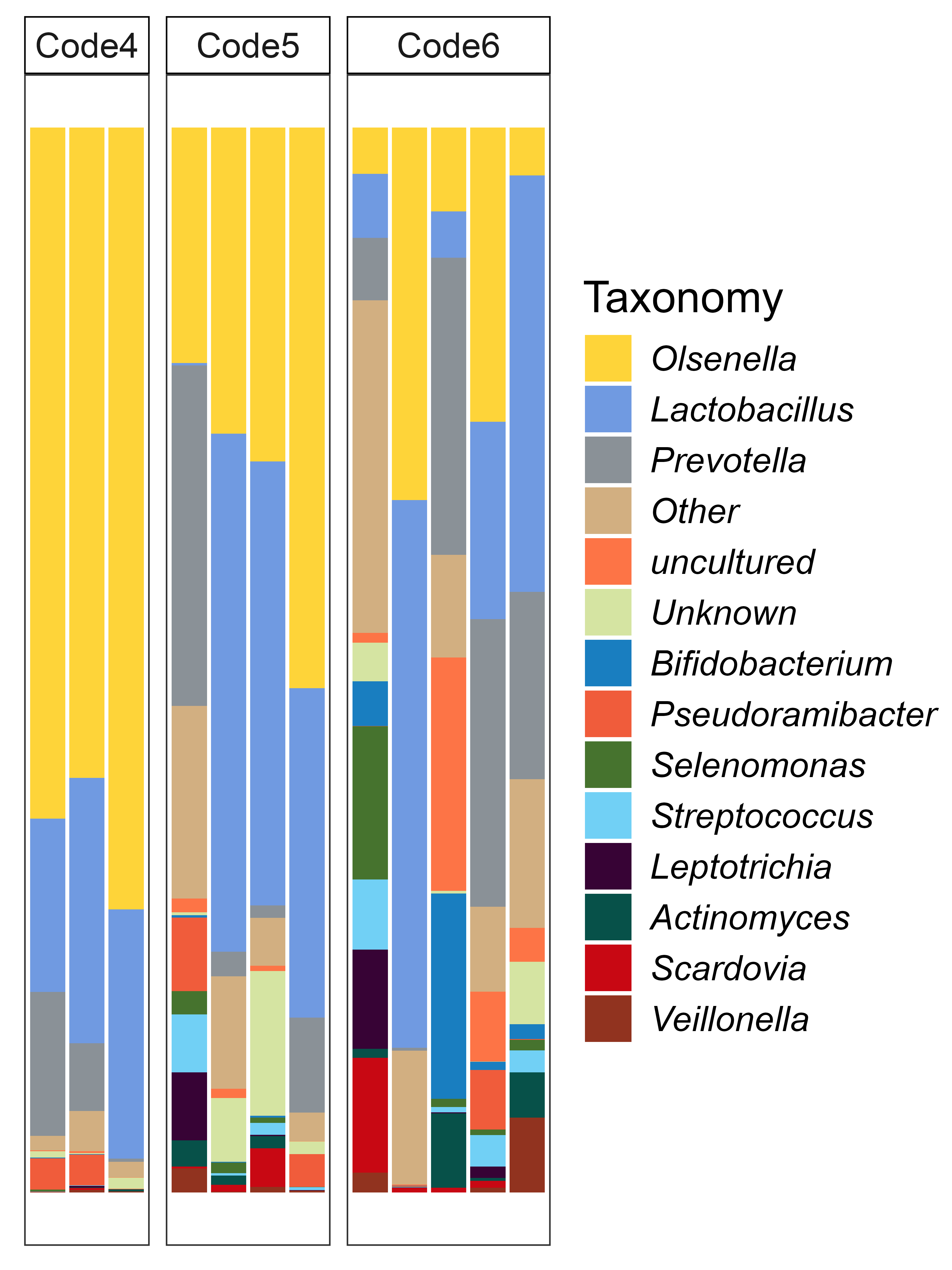


Composition of the oral microbial profile at the genus level as the percentage of relative abundance by the ICDAS code classification. The mean of the relative abundance by each group was shown on the left.
